# Bovine Fetal Mesenchymal Stem Cells Obtained From Omental Adipose Tissue and Placenta Are More Resistant to Cryoprotectant Exposure Than Those From Bone Marrow

**DOI:** 10.3389/fvets.2021.708972

**Published:** 2021-10-04

**Authors:** Rudy Oyarzo, Ximena Valderrama, Francisca Valenzuela, Javiera Bahamonde

**Affiliations:** ^1^Laboratory of Applied Morphology, Instituto de Farmacología y Morfofisiología, Facultad de Ciencias Veterinarias, Universidad Austral de Chile, Valdivia, Chile; ^2^Instituto de Investigaciones Agropecuarias, INIA Remehue, Osorno, Chile; ^3^Department of Biomedical Sciences and Pathobiology, Virginia-Maryland College of Veterinary Medicine, Virginia Tech, Blacksburg, VA, United States; ^4^Escuela de Graduados, Facultad de Ciencias Veterinarias, Universidad Austral de Chile, Valdivia, Chile

**Keywords:** mesenchymal stem cells, fetuses, bovine, cryoprotectants, adipose tissue, bone marrow, placenta

## Abstract

Recent studies have shown promise for the development of cellular therapies with mesenchymal stem cells (MSCs) in livestock species, specifically bovines, and cryopreservation is highly relevant for the advancement of these applications. The use of permeable and/or non-permeable cryoprotectant solutions is necessary to reduce cell damage during freezing and thawing, but these same compounds can also cause negative effects on MSCs and their therapeutic properties. Another important factor to consider is the tissue source of MSCs, since it is now known that MSCs from different tissues of the same individual do not behave the same way, so optimizing the type and concentration of cryoprotectants for each cell type is essential to achieve a large and healthy population of MSCs after cryopreservation. Furthermore, sources of MSCs that could provide great quantities, non-invasively and without ethical concerns, such as placental tissue, have great potential for the development of regenerative medicine in livestock species, and have not been thoroughly evaluated. The objective of this study was to compare the viability of bovine fetal MSCs extracted from bone marrow (BM), adipose tissue (AT), and placenta (PT), following their exposure (15 and 30 min) to several solutions of permeable (dimethyl sulfoxide and ethylene glycol) and non-permeable (trehalose) cryoprotectants. Viability assays were performed with Trypan Blue to assess post-exposure plasma membrane integrity. The apoptotic potential was estimated analyzing the mRNA abundance of BAX and BCL-2 genes using quantitative rt-PCR. Based on the results of the study, BM-MSC exhibited significantly lower viability compared to AT-MSC and PT-MSC, at both 15 and 30 min of exposure to cryoprotectant solutions. Nevertheless, viability did not differ among treatments for any of the cell types or timepoints studied. BCL-2 expression was higher in BM-MSC compared to AT-MSC, however, BAX/BCL-2 ratio did not differ. In conclusion, AT-MSC and PT-MSC were more resistant that BM-MSC, which showed higher sensitivity to experimental conditions, regardless of the exposure times, and cryoprotectant solutions used in the study.

## Introduction

Mesenchymal stem cells (MSCs) have generated great interest as a potential cell population to be used therapeutically and thus become a tool for alternative treatments of various diseases in both humans and animals. In regenerative veterinary medicine, and more specifically in bovines, the knowledge leading toward the therapeutic use of these cells is still very limited when compared to other species, therefore rising the need to fill this gap (1, 2). For all species, MSCs are considered a heterogeneous population of non-hematopoietic progenitor cells derived from the mesoderm with multi-differentiation and self-renewal capacities (3). In humans, it has been well-defined that they must have the ability to adhere to plastic under standard culture conditions, express specific surface antigens CD105, CD73, and CD90 and, at the same time, not to express CD45, CD34, CD14/CD11b, CD79α/CD19, and HLA-DR. Finally, they must be able to differentiate at least into osteoblasts, adipocytes and chondroblasts under *in vitro* conditions (4).

Regenerative veterinary medicine has focused mainly on high performance and companion animals, but recent studies carried out in livestock species could open a field for new applications. Within the domestic species, cattle have an important economic role in the livestock industry, however, there are pathological conditions, such as mastitis, that can negatively affect their productive parameters (5). Mastitis significantly reduces milk production, affects udder tissue, reduces the value of the animal and it is also an animal welfare problem. The damage caused by this disease in the breast tissue cannot be reversed with current treatments, so stem cell therapy could be a new therapeutic option that promotes the regeneration of functional tissue of the udder with minimal side effects and could also decrease the severity of the disease (2, 6). It has been demonstrated that MSCs produce bioactive factors and adhesion molecules, as well as exosomes containing reparative peptides/proteins, mRNA, and microRNA, that help to inhibit scar formation and apoptosis, increase angiogenesis, and stimulate intrinsic progenitor cells to regenerate their functionality, increasing cell proliferation during tissue repair (2, 7). There is also evidence that MSCs can secrete antibacterial peptides capable of inhibiting or hindering the growth of *Staphylococcus aureus, Pseudomonas aeruginosa*, and *Escherichia coli*, etiological agents related to this pathology (8–10). Also, MSCs modified with therapeutic agents could also be used as a possible treatment, since when administered directly to the mammary gland they would promote a strong innate response to this type of infection (6). Hence, therapy with MSC has shown great potential as a complementary treatment for bovine mastitis, but there is still very little information about these cells in cattle.

MSCs can be found in practically all pre and post-natal organs and tissues with multifaceted capacities, collaborating in their repair and support (3). Bone marrow (BM) has been the main source of MSCs for research and clinical trials, however, MSCs have also been isolated from nearly every tissue, including adipose tissue (AT), umbilical cord blood, dental pulp, synovial fluid, and amniotic fluid, among many others. All these tissues present variability in their secreted factors/signals and cellular components, generating different conditions, and physiological niches that can affect the behavior of MSCs, such as their proliferative activity (11, 12).

Bone marrow MSCs (BM-MSCs) were the first cell population described with the singularity of adherence to plastic with fibroblastic morphology capable of generating colonies, and with the previously mentioned therapeutic potential of MSCs. However, its collection from donors is an invasive and painful procedure, in addition to the fact that with age and disease the percentage of MSCs in the BM decreases (2, 3). Therefore, research aims to evaluate other promising sources of MSCs such as AT, which has presented certain advantages including greater ease of isolation, since it is a simple accessible tissue with minimal morbidity for the donor (13, 14). This type of tissue is a less invasive alternative source where greater amounts of MSCs can be obtained compared to BM (15). Another promising source of MSCs is the placental tissue (PT). Being considered a waste tissue, post-partum placenta recollection would not have any repercussion on the donor as it does not require invasive methods for its collection, also is available in great quantities and its use would rise no ethical issues (16–19). In livestock species, it is also possible to obtain early gestation placentas from abattoirs. Despite being a potentially good source of MSCs, few studies are available in domestic animals. One study evaluated canine placenta MSCs as a therapeutic approach for neurological disorders in dogs (20), and another group studied the induction of bovine placental MSCs to differentiate into islet-like cells, with the goal of development of novel therapies for diabetes (17).

It has been shown that MSCs obtained from different tissues can behave differently. In fact, the osteogenic potential of human BM derived MSCs decreases under culture conditions extended over time, unlike the one of AT, which can be maintained *in vitro* for prolonged periods with stable populations and low levels of senescence (3). On the other hand, it has been found that these two cell lines do not have the same ability to overcome adverse conditions. Some studies have shown that AT-MSCs are more resistant to certain factors or variables, such as low concentrations of oxygen (canine MSC), exposure to hydrogen peroxide or serum deprivation (human MSC), as well as certain solutions of cryoprotectants (equine MSC) (21–23). It has even been postulated that AT-MSCs would be less immunogenic by expressing fewer histocompatibility complexes, making them better candidates for allogeneic therapies (24).

In general, one of the most explored alternatives of cell therapy is the use of autologous MSCs, with the difficulty that cells must be isolated and expanded to achieve therapeutic doses. The latter implies a lag time between the moment they are acquired and their use, putting the effectiveness of the treatment at risk. Additionally, with aging and disease the number of cells and their therapeutic properties decrease, therefore, the ideal is to use cells from healthy and young patients (11, 25). Furthermore, MSCs of fetal and placental origin have been found superior than adult MSCs as candidates for allogeneic therapeutic applications, due to their greater proliferative and differentiation capacities, as well as their lower immunogenicity compared with adult MSCs (26–28). The cells can also be harvested, expanded, and stored for later use, facilitating logistics from collection to transplant centers, allowing enough time for their characterization, screening for potential diseases, and application at the exact moment they are needed (3, 29). Taking all this in consideration, the ability of MSCs to survive long periods of storage and at the same time maintain their qualities is critical for the development of allogeneic cell therapies.

If MSCs are *in vitro* cultured for lengthy periods of time, certain risks become significant, such as contamination, phenotypic instability, genotypic variation, and chromosomal alterations (30, 31). Furthermore, it has been demonstrated that their differentiation potential, as well as the number of cells, decrease with culturing time (32). On the contrary, cryopreservation, if properly managed, presents itself as the perfect method to store MSCs for therapeutic purposes. This alternative facilitates quality control, decreases the requirement for fresh tissues and allows standardization of isolation and storage protocols, in order to have a reserve of MSCs that can be used as a reference to validate different assays (33). However, this same process aimed at preserving living cells could also cause damage and compromise their survival, because cells are subjected to structural and molecular changes that can be harmful and could have an impact on their therapeutic applications. Also considering that MSCs from different sources could react differently, this process needs to be thoroughly investigated and optimized for each cell type (3, 34).

Cryoprotectants are used to minimize physical and structural damage of cells during freezing and thawing, with their concentration being one of the most important factors related to the survival of frozen cells (35). These compounds are classified as permeable and non-permeable in terms of their ability to cross the cell membrane and are, in general, low toxicity reagents that reduce cell injuries by minimizing the formation of ice crystals that form both outside and inside of the cell during the freezing process (36).

Permeable cryoprotectants such as dimethyl sulfoxide (DMSO) are compounds that cause dehydration by replacing intracellular water, hence avoiding an excessive concentration of solutes in the extracellular environment and preventing crystal formation inside, with the aim of reducing osmotic stress (3, 37). DMSO is able to solubilize a wide range of poorly soluble polar and non-polar molecules, which together with its low toxicity at concentrations beneath 10% have made it useful for multiple purposes. Indeed, it is one of the most used cryoprotectants due to its low cost and ability to easily penetrate membranes (36, 38). Nevertheless, it has been reported that the use of DMSO could cause alterations in DNA methylation and histones, it has been associated with generating cellular differentiation, and its toxicity at the *in vivo* level has been demonstrated, so its use to preserve MSCs could cause undesirable effects in clinical applications (3, 36, 39, 40). Additionally, a decrease in cell viability and number of colonies has been reported for some cell types cryopreserved with DMSO, and it has been hypothesized that this decrease in cell survival may be related to the apoptosis process which some cells incur when they come in contact with this type of cryoprotectant (3, 34). Ethylene glycol (EG), from the same group of cryoprotectants, is an alternative that has better permeability and less toxicity than DMSO. This compound is commonly used for the cryopreservation of embryos of domestic animals such as rabbits, sheep, and cattle, as well as in vitrification procedures (41, 42).

Non-permeable cryoprotectants can preserve cells at lower molar concentrations than permeable ones, with the disadvantage that they require faster freezing rates to generate protection (43). Trehalose is a sugar within this group that can eliminate water from the cells during the initial phases of freezing, since it is found extracellularly, and thus prevent the formation of ice crystals within the cell (44). Its effects have also been attributed to interactions with lipid membranes, achieving stabilization of proteins during freezing and thawing processes, and also the ability to form a vitreous matrix that can contribute to the inhibition of potentially lethal intracellular ice formation (3, 45). Taking into consideration the mechanism of action, as well as the advantages and disadvantages of each group of cryoprotectants, it is important to evaluate the best combination to obtain better results, such as higher percentages of viability, for instance, when mixing permeable and non-permeable cryoprotectants (3, 46).

There is a large array of research that has evaluated cryopreserved MSCs of different sources using a variety of methods such as cryoprotectants, cooling rates, temperatures, and storage periods (3), and it is known that each cell type exhibits an individual freezing and thawing behavior, thus requiring its specific optimal cryopreservation protocol (47, 48). One of the most important factors in the optimization of cryopreservation is the choice of appropriate cryoprotectants for each cell population. There is very little information regarding effects of cryopreservation and cryoprotectants on bovine MSCs, so far there is only one study that compared the viability of MSCs from rats, mice and bovines when exposed to different types and concentrations of cryoprotectants, in which it was shown that the latter were more sensitive than their counterparts from other species (49), however, the study did not investigate MSC sources other than BM. Information regarding MSCs derived from bovine fetal BM, AT or PT and how they would perform against cryopreservation or cryoprotectants themselves is not currently available.

Understanding the origin and performance of stem cells is essential for multiple potential applications, however, there are still profound gaps regarding the effects of exposure to cryoprotectants and how they affect MSCs that have been obtained from different tissues (2, 34). The main objective of this work was to compare the viability of bovine fetal MSCs extracted from bone marrow, adipose tissue, and placenta, following their exposure to several cryoprotectant solutions. Our hypothesis was that the viability of bovine fetal AT-MSC and PT-MSC would be higher compared to BM-MSC after exposure to different cryoprotectant solutions.

## Materials and Methods

The use of animal samples in this study was reviewed and approved by the Bioethics Committee of the Universidad Austral de Chile (resolution N° 334/2018).

### Isolation and Culture of Bovine Fetal BM-MSC, AT-MSC, and PT-MSC

Late term bovine fetuses and gravid uteri were transported from a local abattoir on the same day of slaughter in a sealed bag inside a plastic container. Fetal BM-MSC were obtained following the protocol by Cortes et al. (50), with minor modifications. Briefly, the diaphyses of both fetal femurs were sectioned and bone marrow was aspirated with previously prepared high glucose Dulbecco's Modified Eagle Medium (DMEM; Corning) supplemented with 1% antibiotic and antifungal (AA; Gibco) syringes. The aspirates were then washed twice with phosphate buffered saline (PBS; Gibco) supplemented with 1% AA, and twice with DMEM + AA. Finally, the samples were seeded with DMEM supplemented with 10% fetal bovine serum (FBS; Gibco) and 1% AA (MSCs medium), and incubated in a 175 cm^2^ culture bottle at 38.5°C with 5% CO_2_ in a humid atmosphere for 36–48 h.

Bovine fetal AT-MSC were harvested following a previously reported protocol (12) with minor modifications. Briefly, samples of omental AT were obtained from the same fetuses as BM-MSC, washed four times with PBS + AA, minced and incubated with 0.5% type 1 collagenase (Gibco) at 37°C for 45 min under shaking. Subsequently, the collagenase was inactivated with MSCs medium and samples were filtered with a 40 μm cell strainer (Falcon). The filtrates were then washed once with DMEM + AA, plated with MSCs medium in a 175 cm^2^ culture bottle and incubated at 38.5°C with 5% CO_2_ in a humid atmosphere for 36–48 h.

PT-MSC were isolated from abattoir derived bovine gravid uteri, using a similar protocol as for AT-MSC. Cotyledon tissue samples were collected from at least 3 different placentomes and washed four times with PBS + AA. Subsequently, the tissue was minced and digested with collagenase (0.5%) at 37°C for 45 min, with shaking. Then, MSC medium was added to inactivate collagenase and digested products were filtered through 40 μm pore cell strainers. Filtrates were washed once with DMEM + AA and finally the cells were resuspended in MSC medium and seeded in a 175 cm^2^ culture bottle, which was incubated at 38.5°C with 5% of CO_2_ in a humid atmosphere for 36–48 h.

All cells were observed daily in order to monitor and compare their adherence to plastic, growth, characteristic fibroblast morphology, and arrangement in the culture bottles. Following the initial 36–48 h of incubation, non-adherent cells in each culture were eliminated by changing the culture medium. Afterwards, media was changed every 48–72 h and cultures were expanded until passage 2 (P2). When at least 90% confluent, P2 cultures were detached with trypsin 0.25% and EDTA 380 mg/L (Gibco), characterized as described below and used for the experiments.

Each fetus was considered one repetition (*n* = 5 and *n* = 6 for BM-MSC and AT-MSC, respectively) and pools of 1–3 gravid uteri obtained within the same day were considered one repetition for PT-MSC (*n* = 6, except where expressly stated). Viability experiments were performed in duplicate for each repetition in order to reduce the variability of the technique used.

### Gene Expression Analysis for Characterization of BM-MSC, AT-MSC, and PT-MSC

In order to validate that cells used correspond to MSCs, the expression of genes characteristic to MSCs were measured; CD73, CD90, CD105 of mesenchymal character, and as negative controls CD34 and CD45 for hematopoietic characters (Table 1).

**Table 1:** Sequences of specific endogenous, mesenchymal, hematopoietic, and apoptotic primers used in qPCR assays.

**Gene**	**Sequence 5** ^**′**^ **-3** ^**′**^		**Length (base pairs, bp)**	**Product length (bp)**
**Endogenous**				
GAPDH	Forward	CCTTCATTGACCTTCACTACATGGTCTA	28	127
	Reverse	TAGAAGATGGTGATGGCCTTTCCATTG	27	
B-ACTIN	Forward	CGCACCACTGGCATTGTCAT	20	227
	Reverse	TCCAAGGCGACGTAGCAGAG	20	
**Mesenchymal**				
CD73	Forward	TGGTCCAGGCCTATGCTTTTG	21	115
	Reverse	GGGATGCTGCTGTTGAGAAGAA	22	
CD90	Forward	CAGAATACAGCTCCCGAACCAA	22	97
	Reverse	CACGTGTAGATCCCCTCATCCTT	23	
CD105	Forward	CGGACAGTGACCGTGAAGTTG	21	115
	Reverse	TGTTGTGGTTGGCCTCGATTA	21	
**Hematopoietic**				
CD34	Forward	TGGGCATCGAGGACATCTCT	20	107
	Reverse	GATCAAGATGGCCAGCAGGAT	21	
CD45	Forward	CCTGGACACCACCTCAAAGCT	21	101
	Reverse	TCCGTCCTGGGTTTTATCCTG	21	
**Apoptotic**				
BAX	Forward	TTGCTTCAGGGTTTCATCCA	21	126
	Reverse	CCGATGCGCTTCAGACACT	19	
BCL-2	Forward	GAGTCGGATCGCAACTTGGA	20	120
	Reverse	CTCTCGGCTGCTGCATTGT	19	

For RNA purification, the Quick-RNA Mini Prep kit (Zymo Research) was used following the manufacturer's instructions. RNA concentration was measured with the Qubit fluorometer (Invitrogen) using the Qubit RNA BR Assay kit (Invitrogen). The RNA samples were subsequently stored at −80°C until further analysis.

Before QPCR gene expression, RNA products were converted to cDNA using the AffinityScript QPCR cDNA Synthesis kit (Agilent Technologies) following the manufacturer's instructions. The reaction (20 μL) was incubated at 25°C for 5 min to allow binding of the primers, then at 42°C for 15 min to allow the synthesis of cDNA and finally at 95°C for 5 min to terminate the reaction by reverse transcriptase denaturation. The cDNA samples were stored at −20°C until later use.

Brilliant II SYBR Green QPCR Master Mix (Agilent Technologies) was used to detect and quantify the transcripts of interest. The cDNA samples were processed in a Quantum Studio 3 Real Time PCR System thermal cycler under the following conditions: 50°C for 2 min, 95°C for 10 min, and 40 cycles at 95°C for 30 s, 60°C for 1 min and 95°C for 15 s, ending with the melting curve step at 95°C for 15 s, 60°C for 1 min and 95°C for 1 s. The data obtained was analyzed with the thermal cycler software: QuantumStudio Design and Analysis v1.4 and Excel version 2002 Microsoft Office 365 ProPlus, using the ΔΔCT formula described by Vandesompele et al. (51). The reference genes GAPDH and B-ACTIN were used to normalize the relative expression of the genes of interest (Table 1). For the characterization of MSCs, the gene expression of mesenchymal markers CD73, CD90, CD105, and hematopoietic markers CD34 and CD45 were analyzed for each cell line (*n* = 5 for BM-MSC and PT-MSC, *n* = 6 for AT-MSC). Each reaction was carried out in triplicate, the three threshold cycles (CT) obtained being averaged. These average values were assigned a percentage of efficiency that, when related to the CT of the control gene and endogenous genes, obtained quantifications of relative expressions for each gene of interest that were then normalized, these being the data presented in the present work (51).

### Exposure to Cryoprotectants

BM-MSC, AT-MSC, and PT-MSC basal cell viability was determined by mixing a sample of cell suspension with Trypan Blue in a 1:1 ratio and analyzing it in duplicate using a Countess II Automated Cell Counter (Invitrogen). Then, each cell suspension was divided and exposed to 5 experimental cryoprotectant solutions. All solutions contained DMEM and FBS, along with one permeable cryoprotectant (DMSO or EG) with or without a non-permeable cryoprotectant, Trehalose, in the concentrations described in Table 2. Cells (1 × 10^6^ live cells/mL) were exposed to the solutions for up to 30 min at room temperature. No longer exposure times were evaluated because prolonged exposure to DMSO is well-known to be cytotoxic and has also been associated with cell differentiation (39). Furthermore, no longer exposure times to cryoprotectants at room temperature are expected during the cryopreservation process since cells usually are promptly started on the freezing protocol after being resuspended with cryoprotectants.

**Table 2:** Composition of experimental cryoprotectant solutions.

G1 (control)	DMEM + FBS (20%)
G2	DMEM + FBS (20%) + DMSO (10%)
G3	DMEM + FBS (20%) + EG (10%)
G4	DMEM + FBS (20%) + DMSO (5%) + Trehalose (5%)
G5	DMEM + FBS (20%) + EG (5%) + Trehalose (5%)

*DMEM, Dulbecco's modified eagle medium; FBS, fetal bovine serum; DMSO, dimethyl sulfoxide; EG, ethylene glycol*.

### Viability Analysis

Samples of each cell suspension were taken to evaluate integrity of the plasma membrane, and thus viability, after 15 and 30 min of exposure. Each sample was mixed with Trypan Blue in a 1:1 ratio and analyzed in duplicate using a Countess II Automated Cell Counter (Invitrogen). Both living and dead cells were counted, and an average percentage of viability was obtained per sample.

### Apoptosis Analysis

In order to assess if apoptosis was involved in the response of MSCs against cryoprotectants, the levels of expression and relation between BAX (pro-apoptotic) and BCL-2 (anti-apoptotic) genes were measured in the tissues with higher and lower viability, after 30 min of exposure to the experimental solutions. The genes studied, primers used, and their products are indicated in Table 1. Samples were processed and analyzed as described in section Gene Expression Analysis for Characterization of BM-MSC, AT-MSC, and PT-MSC.

### Statistical Analysis

The data obtained were analyzed with the program GraphPad Prism version 5.00 for Windows. For the analysis of characterization of the MSCs, as well as the post-exposure viability assessments to the 5 experimental treatments, the ANOVA analysis test was used. In addition, Student's *t*-test was used for the analysis of apoptosis between tissues, using a 95% confidence interval for all cases. All data are expressed as mean ± standard error of the mean (SEM).

## Results

### Characterization of BM-MSC, AT-MSC, and PT-MSC

**Figure 1:**
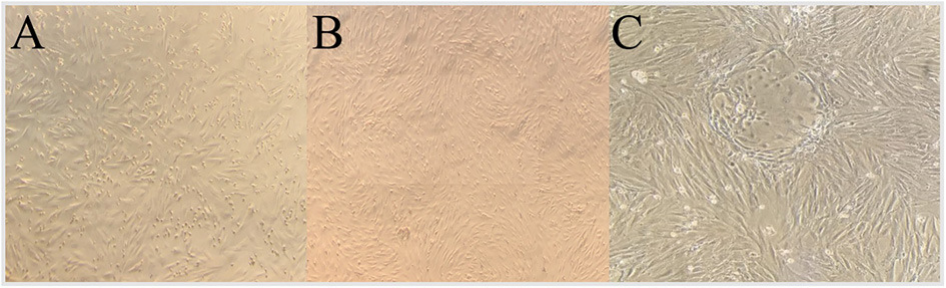
Representative P2 culture photos of bovine fetal AT-MSC **(A)**, BM-MSC **(B)**, and PT-MSC **(C)**, prior to their use for viability assays.

**Figure 2:**
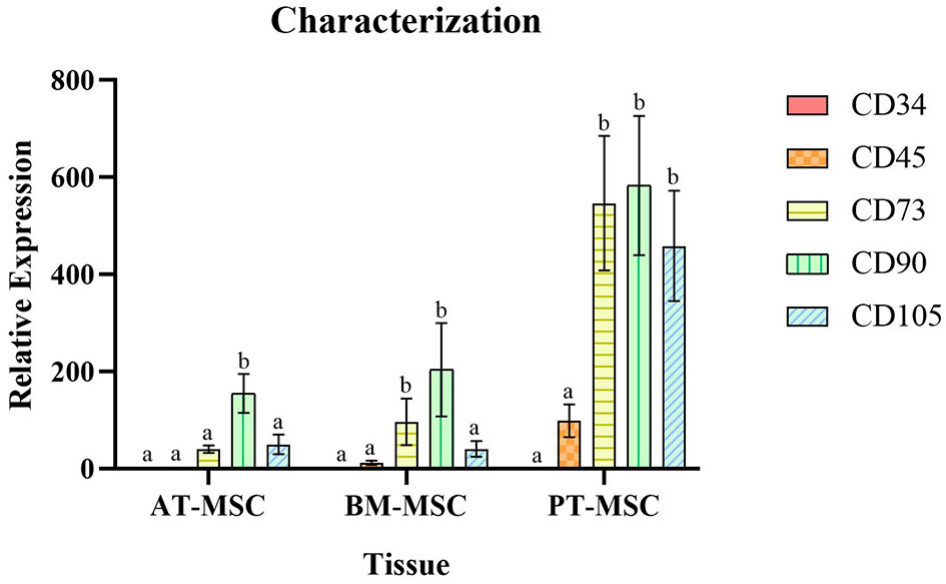
Average relative expression ± SEM of hematopoietic (CD34 and CD45) and mesenchymal markers (CD73, CD90, and CD105) in bovine fetal adipose tissue (AT-MSC), bone marrow (BM-MSC), and placenta (PT-MSC) mesenchymal stem cells. (a, b) Different letters represent significant statistical differences (*P* < 0.05) with respect to CD34 within the same tissue.

**Figure 3:**
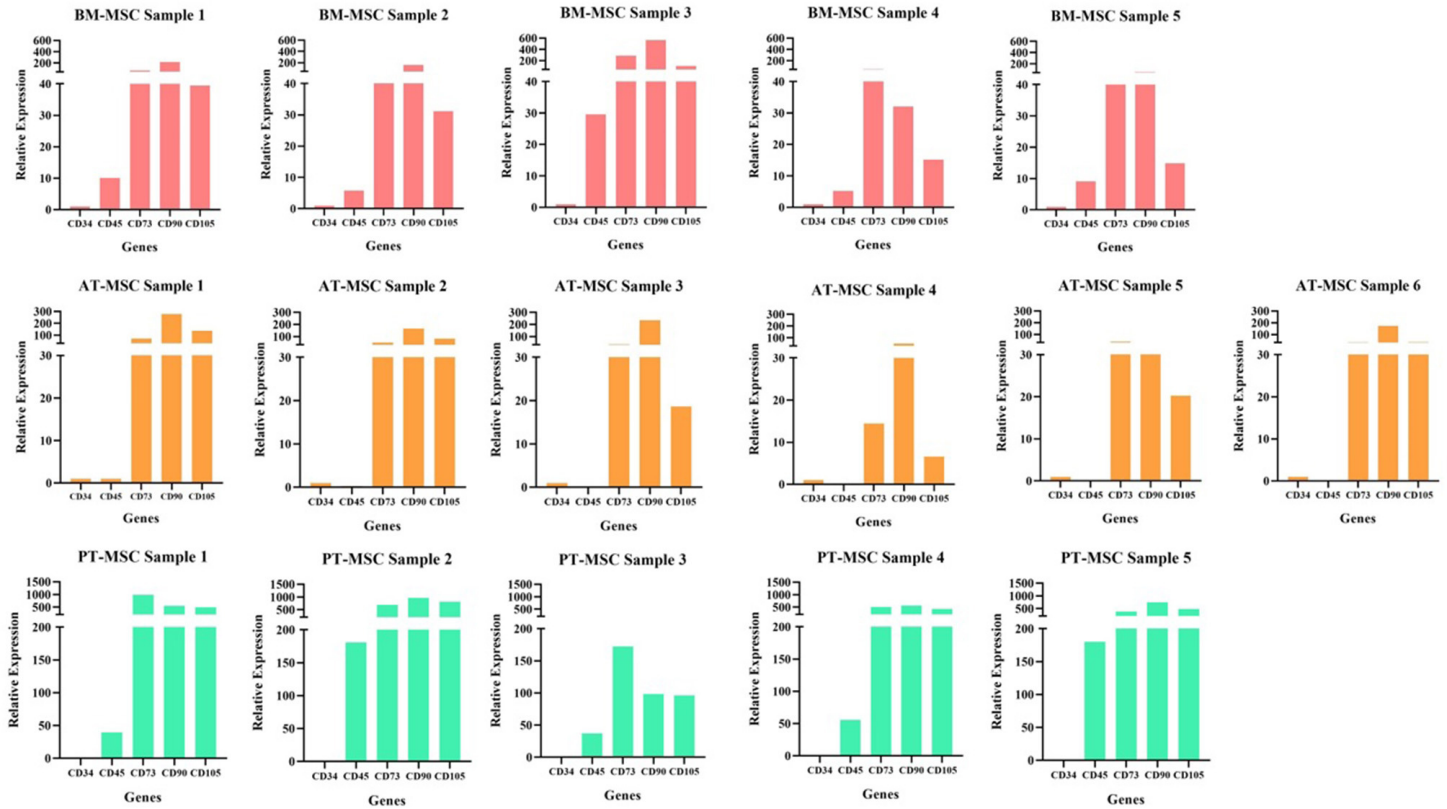
Individual samples relative expression of hematopoietic (CD34 and CD45) and mesenchymal markers (CD73, CD90, and CD105) in bovine fetal adipose tissue (AT-MSC), bone marrow (BM-MSC), and placenta (PT-MSC) mesenchymal stem cells.

All MSC cultures exhibited the adherence to plastic characteristic and spindle shaped morphology of MSCs (Figure 1). In the case of the PT-MSC cultures, some circular structures were occasionally observed as seen in Figure 1C. Characterization of bovine fetal BM-MSC, AT-MSC, and PT-MSC was performed by measuring the relative gene expression of a set of cellular markers recommended by the international society for cell therapy (ISCT). The genes studied, primers used, and their products are indicated in Table 1. To allow comparison between samples, the expression of CD34 was arbitrarily set to a value of 1, and the expression of the other characterizing genes was calculated as expressions relative to CD34, as reported before (24). In general, high levels of gene expression of mesenchymal markers and low levels of gene expression of hematopoietic markers were detected (Figure 2). The mean values of relative expression in relation to CD34 can be seen in Table 3. Significant differences (*P* < 0.05) were detected in CD73 and CD90 for BM-MSC, CD90 for AT-MSC, and CD73, CD90, and CD105 for PT-MSC when compared to CD34 within the same cell type (Figure 2). In all cases, CD73, CD90, and CD105 were expressed at least 14-, 31-, and 6-fold relative to CD34 (Figure 3).

**Table 3:** Mean values of expression of hematopoietic and mesenchymal genes relative to CD34 (mean ± SEM) (a, b) different letters represent significant differences (*P* < 0.05) with respect to CD34 within the same tissue.

**Gene**	**AT-MSC**	**BM-MSC**	**PT-MSC**
CD45	0.2 ± 0.15^a^	12 ± 4.50^a^	99 ± 33.6^a^
CD73	40 ± 7.52^a^	96 ± 47.8^b^	547 ± 138.3^b^
CD90	155 ± 40.0^b^	204 ± 96.1^b^	583 ± 143.1^b^
CD105	50 ± 20.2^a^	41 ± 16.1^a^	459 ± 113.8^b^

### Cell Viability of BM-MSC, AT-MSC, and PT-MSC Post-exposure to Cryoprotectant Solutions

**Figure 4:**
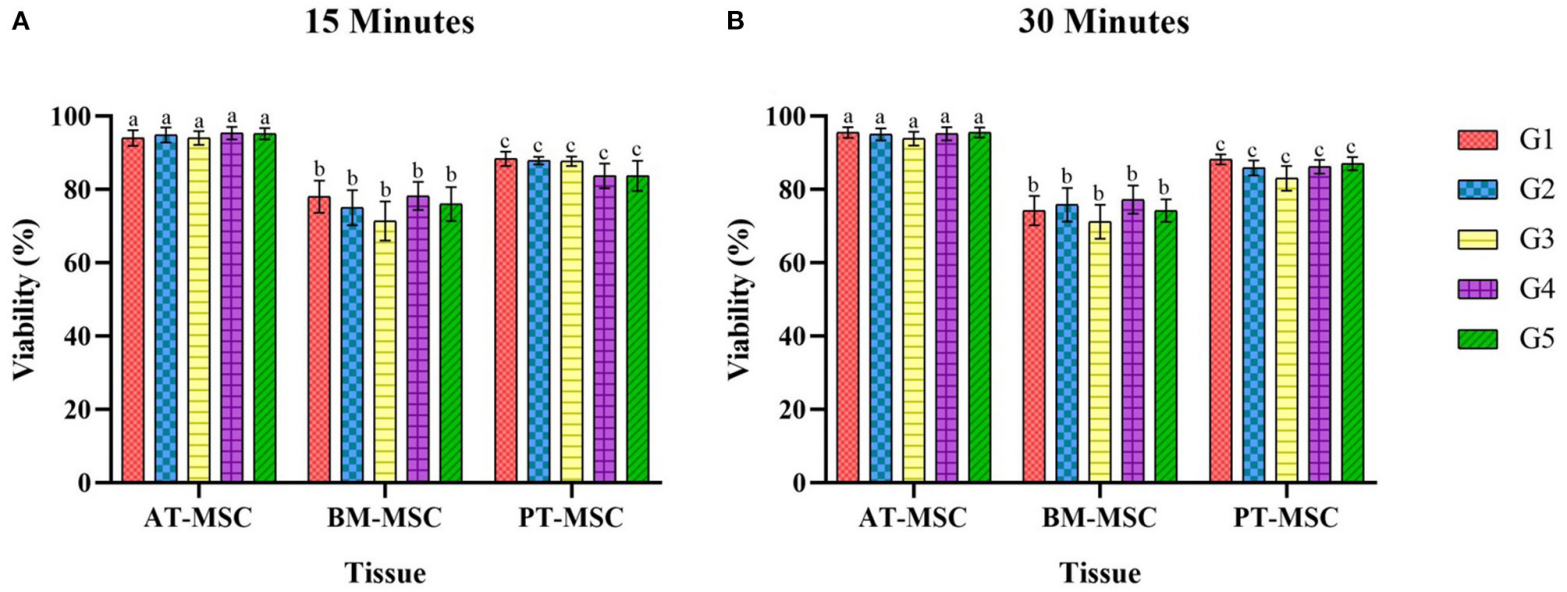
Average viability (%) ± SEM of bovine fetal adipose tissue (AT-MSC), bone marrow (BM-MSC), and placenta (PT-MSC) mesenchymal stem cells exposed to cryoprotectant solutions for 15 and 30 min [**(A,B)**, respectively]. G1 corresponds to the control group without cryoprotectants. (a–c) Different letters indicate significant statistical differences (*P* < 0.001) between groups.

After exposure of the MSCs to the experimental solutions for 15 and 30 min, the data obtained from the count of live and dead cells was analyzed in order to evaluate the ability of MSCs to survive after being in contact with cryoprotectants (average viability percentage). Viability after treatments for 15 min resulted in 94–95.5%, 71.4–78.2%, and 83.6–88.3% for AT-MSC, BM-MSC, and PT-MSC, respectively (Table 4 and Figure 4A). Viability after 30 min of treatment exposure resulted in 93.8–95.5%, 71.2–77.2%, and 83–88.1% for AT-MSC, BM-MSC, and PT-MSC, respectively (Table 4 and Figure 4B). Although there were no statistically significant differences between the different treatments and exposure times studied, there were important differences between tissue sources studied (*P* < 0.001; Table 4 and Figure 4).

**Table 4:** Mean percentages of viability of AT-MSC, BM-MSC, and PT-MSC after 15 and 30 min of exposure to cryoprotectant solutions (mean ± SEM) (a–c) different letters indicate significant differences (*P* < 0.001) between the tissues studied.

	**AT-MSC**	**BM-MSC**	**PT-MSC**
**15 min**			
G1	94.0 ± 2.1%^a^	78.0 ± 4.3%^b^	88.3 ± 1.9%^c^
G2	94.8 ± 2.0%^a^	75.0 ± 4.8%^b^	87.8 ± 1.0%^c^
G3	94.0 ± 1.8%^a^	71.4 ± 5.3%^b^	87.6 ± 1.3%^c^
G4	95.3 ± 1.7%^a^	78.2 ± 3.8%^b^	83.6 ± 3.3%^c^
G5	95.5 ± 1.5%^a^	76.0 ± 4.5%^b^	83.6 ± 4.1%^c^
**30 min**			
G1	95.5 ± 1.4%^a^	74.2 ± 4.0%^b^	88.1 ± 1.3%^c^
G2	95.0 ± 1.6%^a^	75.8 ± 4.5%^b^	85.8 ± 2.0%^c^
G3	93.8 ± 1.9%^a^	71.2 ± 4.6%^b^	83.0 ± 3.3%^c^
G4	95.1 ± 1.7%^a^	77.2 ± 3.8%^b^	86.1 ± 1.8%^c^
G5	95.5 ± 1.3%^a^	74.2 ± 3.0%^b^	87.0 ± 1.7%^c^

**Figure 5:**
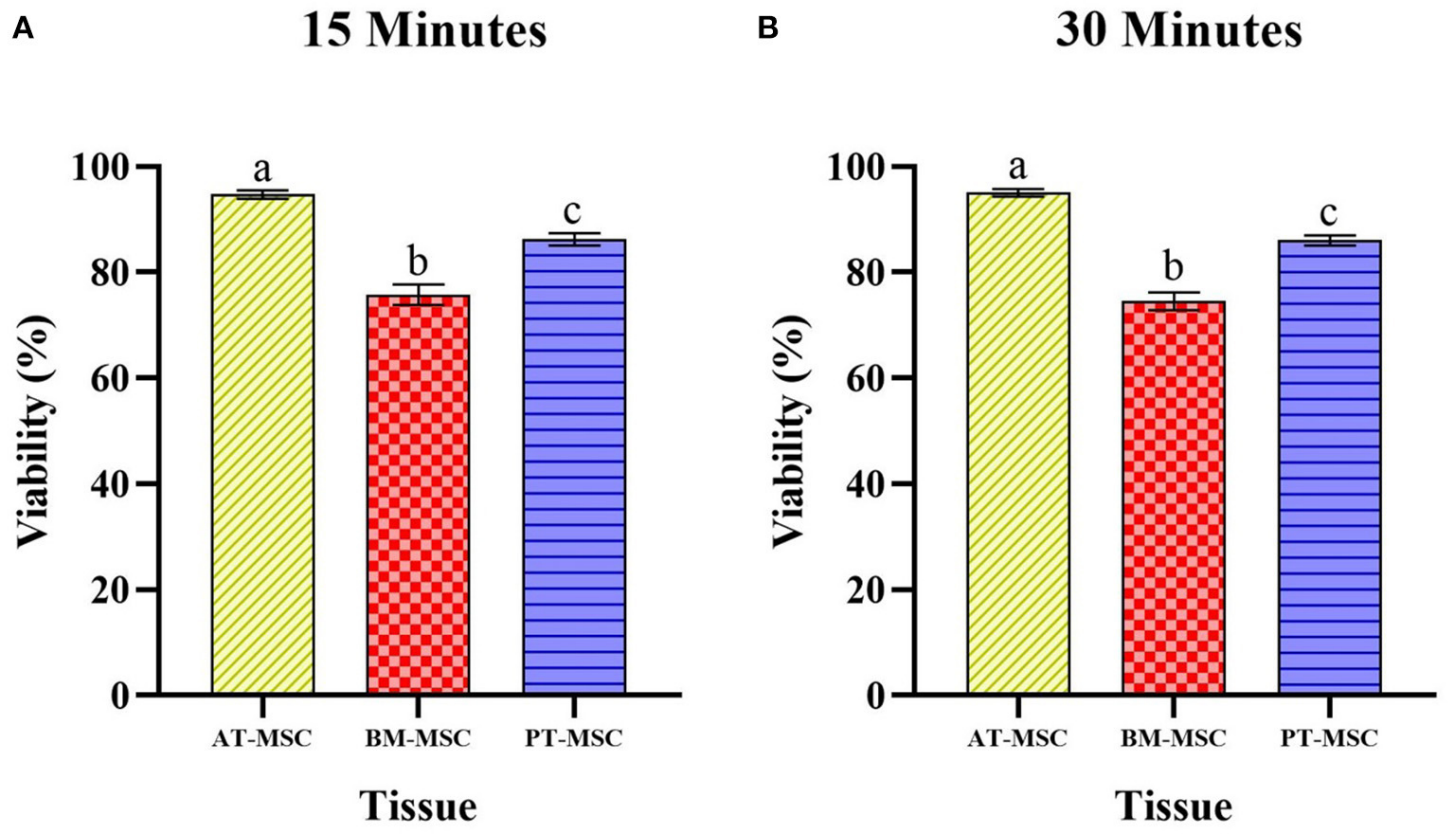
Average viability (%) ± SEM presented by bovine fetal adipose tissue (AT-MSC), bone marrow (BM-MSC), and placenta (PT-MSC) mesenchymal stem cells after being exposed to the experimental treatments for 15 min **(A)** and 30 min **(B)**. (a–c) Different letters indicate significant statistical differences (*P* < 0.001) between the tissue sources studied.

Since no differences were detected between cryoprotectants within a type of MSC, data was compared between tissues observing statistical differences (*P* > 0.0001) among MSC tissue sources. Viability for AT-MSC at 15 min (94.67 ± 0.79%) and at 30 min (95 ± 0.67%) was higher when compared to PT-MSC (15 min 86.23 ± 1.17% and 30 min 86.03 ± 0.96%) and BM-MSC (15 min 75.72 ± 1.95% and 30 min 74.52 ± 1.71%), and in turn, PT-MSC viability was higher than that of BM-MSC at both timepoints considered (Figure 5).

### Apoptotic Potential of BM-MSC and AT-MSC Post-exposure to Cryoprotectant Solutions

**Figure 6:**
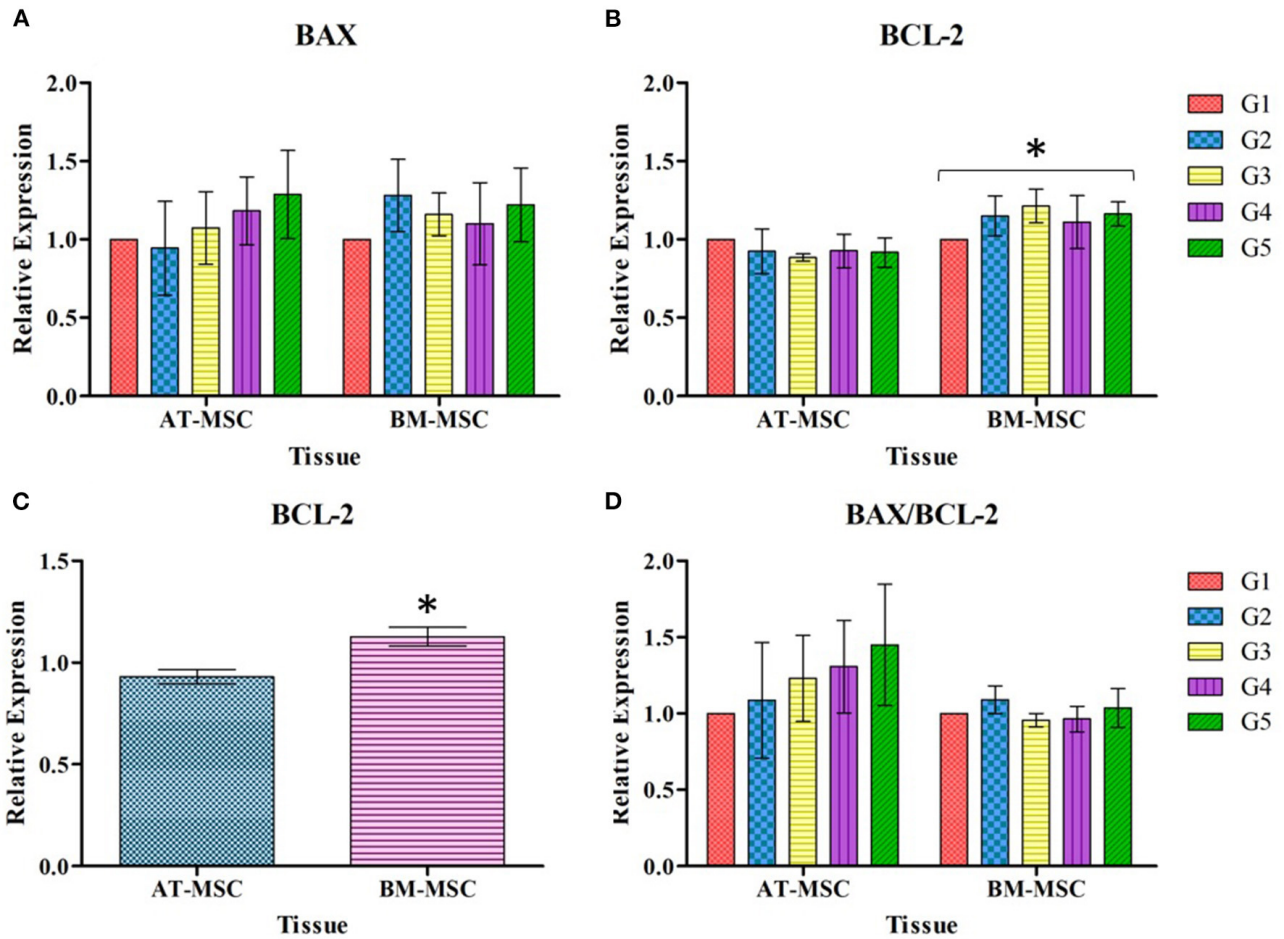
Mean ± SEM relative expression of genes BAX **(A)**, BCL-2 **(B)**, BCL-2 grouped by tissue **(C)**, and BAX/BCL-2 expressed as ratio **(D)**, in AT-MSC and BM-MSC exposed to cryoprotectant solutions for 30 min. *Significant statistical differences (*P* < 0.01) among the tissues studied.

Regarding the relative expression of genes associated with apoptotic response after 30 min of exposure to different cryoprotectant solutions, there were no significant differences in the relative expression of the pro-apoptotic gene BAX in relation to experimental treatments or tissue source (Figure 6A). On the other hand, significant differences (*P* < 0.01) were found for the relative expression of the anti-apoptotic gene BCL-2 between BM-MSC and AT-MSC (Figure 6B), with BM-MSC exhibiting higher expression, but not associated with the cryoprotectant solutions. As there were no differences between the cryoprotectant solutions studied, BCL-2 expression data were grouped and analyzed exclusively by tissue, to highlight the statistical difference (*P* < 0.01) between AT-MSC and BM-MSC (Figure 6C). There were no differences for the BAX/BCL-2 ratio between tissues nor treatments (Figure 6D).

## Discussion

The most striking characteristics of MSCs are their multiple properties of differentiation and immunomodulation, which open a wide range of therapeutic alternatives. To meet those expectations, it is necessary to cryopreserve them, but this same process and the agents used to protect them can cause certain changes in cells and their behavior, effects that could vary according to the tissue from which they were isolated (3). In the present work, viability was evaluated in bovine fetal AT-MSC, BM-MSC, and PT-MSC after exposure to different experimental solutions of cryoprotectants, to elucidate differences in cell mortality among cryoprotectant solutions and/or among tissue sources studied.

To characterize the cells, the relative gene expression of hematopoietic (CD34 and CD45) and mesenchymal markers (CD73, CD90, and CD105) was investigated (Figure 2). In general, the cells expressed high levels of mesenchymal markers and comparatively low levels of hematopoietic markers, but we observed high variability in the levels of expression among individuals (Figures 2, 3). Within BM-MSC, CD73, and CD90 presented significantly higher expressions compared to CD34, while within AT-MSC, only CD90 expression was significantly higher when compared to CD34. The absence of statistical significance for some of the genes in BM-MSC and AT-MSC is probably due to the high individual variability in the levels of gene expression of the mesenchymal markers, but all individuals presented CD73, CD90, and CD105 expressions at least 14-, 31-, and 6-fold relative to CD34, respectively, as shown in Figure 3. For PT-MSC, a clear pattern of expression of mesenchymal markers was observed with CD73, CD90, and CD105 showing significantly higher expressions than CD34 (Table 3 and Figures 2, 3). Peng et al. (17), reported a clear expression of CD73 and not CD45 in stem cells extracted from bovine placenta. Our PT-MSC cultures presented relatively low expression of CD45, but this expression level was not significant compared to CD34. It is important to highlight that the expression of surface markers to characterize MSCs has been well-defined for human cells, but to date, there is no consensus for other species. In addition to this pattern of gene expression, all MSCs were able to adhere to the base of the culture bottles and presented a fibroblastic morphology characteristic of this cell phenotype (Figure 1), thus confirming that the cells isolated and used for this study were effectively MSCs. Similar findings in terms of the defining characteristics of the MSCs were reported in the studies carried out by Huaman et al. (24) and Jervis et al. (12), who also used bovine fetal AT-MSCs and BM-MSCs. Huaman et al. (24) characterized the MSCs by analyzing the expression of the same genes evaluated in the present study, reporting high levels of expression of mesenchymal markers and low levels of expression of hematopoietic markers. Jervis et al. (12), for their part, described characteristics similar to those mentioned above regarding the ability to adhere to plastic and fibroblast morphology in both tissues, which supports the conclusion that the cultures used correspond to MSCs. This pattern also can be visualized in other species, as in the study by Ertas et al. (22), who isolated and cultured human MSCs from BM and AT, exhibiting similar behavior.

Throughout the study, no differences in cell viability were found among the five cryoprotectant solutions tested, so it could be deduced that none of them were more cytotoxic than their counterparts (Figure 4). These results are contrary to those published by some authors such as Liu et al. (46), who cryopreserved human BM-MSC for 1 week with different experimental solutions of cryoprotectants, showing that a solution with 5% DMSO, 2% polyethylene glycol, 3% trehalose, and 2% albumin had superior results when compared to the commonly used 10% DMSO. This could indicate that when the latter is combined with a non-permeable cryoprotectant such as trehalose (also evaluated in this study) would have a beneficial effect on cell viability. However, these authors not only exposed cells to cryoprotectants, but also cryopreserved them at −80°C, so the interaction of the cells with the cryoprotectant solutions was under different conditions from those of the present study. On the other hand, it has been observed that DMSO at lower concentrations (0.1%) produces cellular toxicity *in vivo*, causing significant levels of retinal apoptosis (40), therefore other alternatives to DMSO should be selected when possible for the development of cellular therapies, even if no differences in viability are observed *in vitro*. In any case, further studies are required to evaluate the effectiveness of the cryoprotectant solutions tested here to protect bovine fetal MSCs during freezing and thawing.

Although no significant differences were found associated to the cryoprotectants used nor the time of exposure, MSC viability after exposure to cryoprotectant solutions was different for the three tissue sources studied. In the case of BM-MSC it was around 70%, compared to AT-MSC, whose percentages of viability were around 90% or PT-MSC whose percentages were around 80%, both at 15 and 30 min (Figure 5). These results point out BM-MSC as a cell line more sensitive to external agents than its counterparts. This difference may be due to intrinsic factors of the tissue, as Davies et al. (34) explain when supporting the hypothesis that AT is a source of more robust MSCs. Their study compared cell cryopreservation of rat MSCs derived from AT, BM, and MSC-like cells derived from dental pulp. They described pre-freezing viabilities >95% for all tissues and diminished viabilities after cryostorage, with BM-MSC presenting the lowest post-freezing viability (57%). In addition, after the cryopreservation process, they conducted a study of mesenchymal markers to identify and quantify the presence of this cellular phenotype with respect to the total of cells and thus evaluate if there were variations in the tissues studied. The results revealed that in both, AT-MSC and dental pulp, there was an increase in the expression of these markers, thus affirming that a sort of mesenchymal cell selection took place: since being more resistant they predominated in relation to the total of cells affected by cryopreservation. However, this was not the case for BM-MSC, where there was a lower expression of mesenchymal markers after freezing, leading to the hypothesis that the decrease in viability was not only due to the death of hematopoietic cells, but also of the MSCs, corroborating the idea that BM-MSCs are more susceptible to damage when compared to their counterparts from other tissue sources. In another study carried out by Renzi et al. (23), post-freezing viability of equine MSCs from BM and AT was evaluated using 15 different cryopreservation solutions. They observed that BM-MSCs had consistently lower resistance to intracellular damage caused by ice crystals, regardless of the cryoprotectant used. A similar conclusion was reported by Peng et al. (52) who investigated the cell differentiation potential and the reaction of rat MSCs from BM, AT, and cartilage to hydrogen peroxide exposure and to the deprivation of serum. They demonstrated, on the basis of growth curves, cell cycle, and telomerase activity analysis, that AT-derived cells possessed greater proliferative potential than cells derived from BM and cartilage. These findings coincide with what was discovered in the present study, and they reinforce the claim that AT-MSCs are more resistant than BM-MSCs. Regarding PT-MSC, there is no previously reported information about their reaction to cryopreservation or cryoprotectants, but our results suggest that they are more resistant than BM-MSCs. This theory is supported by a study by Huang et al. (53), that demonstrated resistance of human PT-MSC to adverse conditions such as hypoxia and serum deprivation. Nevertheless, considering there are substantial differences in the placenta of different mammals, further studies are needed to confirm the resistance of ruminant PT-MSCs to hostile conditions or challenges.

Along with the viability, the apoptotic potential (differential sensitivity of cells to apoptotic stimuli) was evaluated in order to elucidate whether certain proteins associated with this process of cell death modified their gene expression when MSCs were exposed to cryoprotectants for 30 min. For this analysis, only two of the three MSC lines were considered, selecting the ones with highest and lowest viability (AT-MSC and BM-MSC, respectively) as an initial screening to clarify whether this difference in the percentages of viability could be explained by the activation of intrinsic mechanisms of cell apoptosis (Figure 6). Increased expression of pro-apoptotic genes is something to be expected in these circumstances, since according to some authors as Bissoyi and Pramanik (54) cryoprotectants induce the release of apoptotic proteins, which is related to the effects produced by these compounds such as changes in osmolarity, that lead to an increase in reactive oxygen species and lesions in the cellular structure and in organelles such as mitochondria, producing cellular stress (55). BAX and BCL-2 are two proteins that play a key role in the regulation of the apoptotic process and have been pointed out as good indicators of the apoptotic potential of cells (56, 57). The first is a pro-apoptotic protein that triggers the process when faced with certain stimuli, interacting with the voltage-gated anion channel located in the outer mitochondrial membrane. This bond activates the formation of a pore which causes the loss of transmembrane potential and the release of cytochrome C into the cytoplasm. The second protein, BCL-2, antagonizes the aforementioned effects by obstructing this bond between BAX and the channel, thus preventing subsequent events. If this obstruction does not occur, free cytochrome C in the cytoplasm binds to APAF-1 and apoptosis occurs. Therefore, it is important to evaluate BAX and BCL-2 as a whole, since only then they can serve as indicators to determine the sensitivity of cells to apoptotic stimuli (54, 57, 58). To estimate and compare MSCs apoptotic potential, in the present study we assessed the relative expression of BAX and BCL-2 genes, as well as the relation between them (BAX/BCL-2). There were no significant differences in the apoptotic response caused by cryoprotectants or by the tissue source in the case of BAX. However, BCL-2 presented higher expression in BM-MSC compared to AT-MSC independent of the cryoprotective solutions. This could be due to the increased sensitivity of the first tissue to external factors, which could trigger the expression of this protein in an effort to avoid the apoptosis process. As Li et al. (59) mention, the BCL-2 protein acts as a critical regulator in this process, inhibiting programmed cell death mechanisms. However, the BAX/BCL-2 ratio must be taken into even greater consideration since, being antagonistic proteins in their functions, their relationship is more important at the time of interpreting the significance of an altered expression of these genes. Analysis of the BAX/BCL-2 ratio yielded no significant differences between BM-MSC and AT-MSC under all the experimental conditions evaluated, so it could be concluded that none of the variables considered generated a physiologically relevant change in the apoptotic potential of these MSCs after exposure to cryoprotectants. Even so, more studies are needed to corroborate whether longer exposure times and/or the process of cryopreservation would change the pattern of expression of apoptotic genes in some, or all, of these tissues.

The absence of relevant statistical differences presented in both, viability and apoptosis, between the different experimental treatments evaluated may be related to the exposure times selected, with 15 and 30 min being a reduced time frame to detect inequalities between treatments and their interaction with MSCs. However, longer exposure times at room temperature are not expected in cryopreservation protocols, when cells are suspended in cryoprotectant solutions and rapidly initiate their freezing process. On the other hand, significant differences between tissue sources in viability, but not in apoptotic potential is an apparent contradiction that arose during the analysis of the study. However, it should be taken into consideration that apoptosis is not the only mechanism of cell death and that it requires time to manifest itself, unlike cell necrosis, which is immediate. The latter may be the result of variables such as mechanical damage during handling, and it is possible that BM-MSCs are prone to suffer more damage during the mere manipulation of cells during standard laboratory procedures, which would explain the apparent incongruity between viability and apoptotic potential results. All of the above is relevant if MSCs are projected to be used for treatments or therapies, where the cells obtained need to suffer as little damage as possible by environmental variables and handling, thereby favoring the more resistant types of MSCs.

In conclusion, the cells used in this study corresponded to MSCs as demonstrated by their gene expression pattern of hematopoietic and mesenchymal markers, ability to adhere to plastic and fibroblast morphology. Bovine fetal AT-MSCs and PT-MSCs present greater resistance to death than BM-MSCs, as evidenced by consistently higher percentages of viability post-exposure to different cryoprotectant solutions. On the other hand, AT-MSCs demonstrated superior viability than PT-MSCs, but the latter have the comparative advantage of coming from a readily available tissue usually considered waste, with no ethical concerns associated. Also, there were no significant differences in apoptotic potential between AT-MSC and BM-MSC, estimated by BAX/BCL-2 gene expression ratio. Taking all this into consideration, AT-MSCs and PT-MSCs are presented as suitable candidates for the development of cell therapies in cattle, and future work should focus on elucidating their response to cryopreservation in terms of viability and also regarding the maintenance of their therapeutic properties.

## Data Availability Statement

The raw data supporting the conclusions of this article will be made available by the authors, without undue reservation.

## Ethics Statement

The animal study was reviewed and approved by the Bioethics Committee of the Universidad Austral de Chile (resolution N° 334/2018).

## Author Contributions

RO participated in data collection, analysis, and preparation of the manuscript. XV participated in data collection, PCR assays, and editing of the manuscript. FV participated in data collection and editing of the manuscript. JB participated in study design, data collection, analysis, and editing of the manuscript. All authors contributed to the article and approved the submitted version.

## Funding

This research was funded by Universidad Austral de Chile (Project DID Regular S-2018-04 and Escuela de Graduados Facultad de Ciencias Veterinarias) and the National Agency for Research and Development (ANID, Project FONDECYT 11180681).

## Conflict of Interest

The authors declare that the research was conducted in the absence of any commercial or financial relationships that could be construed as a potential conflict of interest.

## Publisher's Note

All claims expressed in this article are solely those of the authors and do not necessarily represent those of their affiliated organizations, or those of the publisher, the editors and the reviewers. Any product that may be evaluated in this article, or claim that may be made by its manufacturer, is not guaranteed or endorsed by the publisher.
